# Genomic epidemiological characteristics of dengue fever in Guangdong province, China from 2013 to 2017

**DOI:** 10.1371/journal.pntd.0008049

**Published:** 2020-03-03

**Authors:** Bangyao Sun, Xin Zhang, Huan Zhang, Haizhou Liu, Lina Sun, Qiqi Tan, Mifang Liang, De Wu, Di Liu

**Affiliations:** 1 CAS Key Laboratory of Special Pathogens and Biosafety, Wuhan Institute of Virology, Chinese Academy of Sciences, Wuhan, China; 2 Computational Virology Group, Center for Bacteria and Viruses Resources and Bioinformation, Wuhan Institute of Virology, Chinese Academy of Sciences, Wuhan, China; 3 University of Chinese Academy of Sciences, Beijing, China; 4 Institute of Microbiology, Guangdong provincial Center for Disease Control and Prevention, Guangzhou, China; 5 National Institute for Viral Disease Control and Prevention, Chinese Center for Disease Control and Prevention, Beijing, China; Duke-NUS GMS, SINGAPORE

## Abstract

Dengue fever, a mosquito-borne viral disease in humans, has been endemic in many Southeast Asian countries. Since its first outbreak in 1978 in Foshan, Guangdong province, China, dengue has been continually epidemic in recent years in Guangdong, which raised the concern whether dengue infection is endemic in Guangdong. In this study, we performed phylogenetic, recombinant, and nucleotide variation analyses of 114 complete genome sequences of dengue virus serotypes 1–4 (DENV1-4) collected from 2013 to 2017 in 18 of 21 cities of Guangdong. Phylogenetic analyses revealed that DENV sequences did not form a single cluster, indicating that dengue fever was not endemic in Guangdong, although DENV1-4 co-circulated in Guangdong. Twenty intra-serotype recombinant isolates involving DENV1-4 were detected, but no inter-serotype recombinant events were identified in this study. Additionally, the most recombinant events were detected simultaneously in the gene *NS3* of DENV1-4. Nucleotide variation analyses showed that no significant intra-serotype differences were observed, whereas more significant inter-subtype differences were discovered in non-structural genes than in structural genes. Our investigation will facilitate the understanding of the current prevalent status of dengue fever in Guangdong and contribute to designing more effective preventive and control strategies for dengue infection.

## Author summary

In 1978, dengue fever was first reported in Guangdong province, China, and this has been continuously prevalent in Guangdong in recent years. This is responsible for the heavy burden on the control of dengue, and raises the concern about whether dengue outbreaks have become endemic in Guangdong. Previous studies based on single *E* gene or few full-length genome sequences were inconclusive. In this study, we sequenced 114 DENV complete genomes of DENV1-4 obtained from 2013 to 2017 in Guangdong and further analyzed the epidemiological and molecular characteristics. Phylogenetic analyses revealed that dengue fever was not endemic in Guangdong, which was indirectly supported by results of our recombination analyses. Nucleotide variation analyses indicated that purification selection shaped dengue virus population. Our investigation will facilitate the development of more effective epidemiological surveillance strategies for dengue infection.

## Introduction

Dengue fever was listed as a major threat to global health in 2019 by the World Health Organization, aiming to reduce half of the deaths resulting from dengue infection by 2020 (WHO, https://www.who.int/emergencies/ten-threats-to-global-health-in-2019). Dengue infection is a mosquito-borne disease widely distributed in tropical and subtropical countries with its clinical symptoms ranging from mild dengue fever to severe dengue hemorrhagic fever (DHF) or dengue shock syndrome (DSS)[1]. Approximately two-fifths of the world population are under threat of dengue infection with nearly 390 million infections annually, and it is mainly distributed in Southeast Asia, Africa, Eastern Mediterranean, Americas, and Western Pacific [2].

Dengue fever is caused by dengue virus (DENV), belonging to the genus *Flavivirus* of family *Flaviviridae*. It is primarily transmitted by *Aedes aegypti* or *Aedes albopictus* mosquitoes [3] and is serologically classified into four serotypes (DENV1-4) [4, 5]. The genome of DENV is a single-strand positive-sense RNA of approximately 11 kb, consisting of a single open reading frame that encodes three structural proteins (C, M, and E) and seven nonstructural (NS) proteins (NS1, NS2A, NS2B, NS3, NS4A, NS4B, and NS5), which are flanked by 5’ and 3’ untranslated regions (UTRs) [6].

In China, dengue disease was first recorded in Foshan, Guangdong Province in 1978. Since then, sporadic dengue fever epidemics has occurred in Hainan, Guangxi, Fujian, Zhejiang, and Yunnan provinces in mainland China [7–9]. Particularly, dengue infection is reported almost annually in Guangdong in recent years, contributing to more than half of dengue fever cases in China [10]. In 2014, an unprecedented dengue fever outbreak occurred in Guangdong Province, leading to more than 40,000 infective cases, although great efforts were made to prevent and control dengue infection[11, 12]. In subsequent years, hundreds of dengue cases at least per year have been observed in Guangdong (Table 1, data from Guangdong provincial Center for Disease Control and Prevention). Continuous dengue epidemics have again raised the concern about whether dengue outbreaks have become endemic in Guangdong.

**Table 1:** Dengue cases from 2013 to 2017 in Guangdong Province.

Year	2013	2014	2015	2016	2017
Dengue cases	2894	45189	1683	544	1662

In the current study, we sequenced 114 full-length genomes of DENV1-4 from 2013 to 2017, involving 18 of 21 cities in Guangdong Province and we further performed phylogenetic, recombinant, and nucleotide variation analyses. Our analyses revealed that dengue fever was not endemic in Guangdong but it is still necessary to strengthen the survey of dengue disease. By this investigation, we aimed to provide further support for the control of dengue fever in Guangdong Province.

## Methods

### Ethics statement

This study was performed in accordance with the Declaration of Helsinki and was approved by the ethics committee of Wuhan Institute of Virology. All the patients provided written informed consents.

### Samples collection and virus isolation

The patient's history of travelling was obtained through inquiry. Patients with DENV who did not leave Guangdong within two weeks prior to the onset of the disease were classified as local cases; otherwise classified as imported cases. After collecting serum from patients, type-specific monoclonal anti-DENV bodies were used to identify the serotype by indirect immunofluorescence. DENV positive samples were further used for viral isolation by inoculation into C6/36 cell lines. When cytopathic effect was clearly observed, cells were centrifuged to remove any debris and supernatant were collected and stored at ˗80°C.

### RNA extraction, RT-PCR, and Sanger sequencing

Viral RNA was extracted from 200 μL of cell supernatant using Viral DNA/RNA Extraction kit (QIAGEN, Germany) according to the manufacturer’s instructions. One-step RT-PCR kit (TaKaRa, Japan) was used to amplify DENV sequences according to the manufacturer’s protocols. PCR primer sequences of DENV are provided in S1 Table. Sanger sequencing (ABI 3730) was performed to obtain DENV sequences after PCR products purification (Qiagen, Germany). Sequences assemblies were completed using BioEdit (version 7.1.3.0).

### Dataset collection

We sequenced 114 whole genomes in this study (DENV-1 = 52, DENV-2 = 30, DENV-3 = 23, and DENV-4 = 9). Each analyses dataset of four DENV serotypes was composed of complete genome sequences in this study and those possessing the location and year of isolation available in GenBank database (up to December 31, 2018). Each complete sequence of DENV1-4 was firstly performed through BLASTN analyses and the top 50 hit complete sequences were selected. Next, top hit sequences were combined into a dataset, along with published complete genome sequences isolated in Asia from 2012 to 2017. Sequences with duplicated accession numbers, poor quality, and positive recombinant signal (see “Evidence of recombination”) were excluded from dataset. Finally, four separated datasets (527 DENV-1, 314 DENV-2, 154 DENV-3, and 110 DENV-4 genome sequences) were collected for subsequent analyses. All published DENV complete genome accessions involved in this study are supplied in S2 Table.

### Phylogenetic analyses

Sequences were aligned using ClustalO [13] with subsequent manual correction by BioEdit (version 7.1.3.0). For phylogenetic analyses, UTR sequences were removed. Maximum likelihood (ML) phylogenetic trees were constructed using online web server (CIPRES) [14], as implemented in RAxML [15] program (version 8.2.10), under the General Time Reversible (GTR) model of nucleotide substitution with 1,000 bootstrap replicates. The final ML trees were visualized using Figtree (version 1.4.3).

### Evidence of recombination

Recombination detection was performed using Recombination Detection Program 4 (RDP4) [16] with UTR sequences eliminated. Seven methods were selected, including RDP [16], GENECONV [17], BootScan [18], MaxChi [19], Chimaera [20], SiScan [21], and 3Seq [22]. The recombinant event was determined when recombinant signal was detected by at least two methods and *p* value < 0.05 was considered positive recombinant signal. Detailed detection information of the recombinant isolates is shown in S3 Table.

### Single Nucleotide Polymorphism (SNP) analyses

The consensus sequences of dataset were obtained via cons command in EMBOSS package [23], and SNPs were directly called by aligning the complete genome sequences in this study to the consensus sequence using homemade PERL script (available at https://github.com/zer0liu/bioutils/tree/master/snp).

### Accession numbers

All dengue complete genomes generated in this study were submitted to NCBI GenBank database with accessions no. MN018285-MN018398. Detailed information is supplied in S4 Table.

## Results

### Sequence characteristics in this study

**Fig 1:**
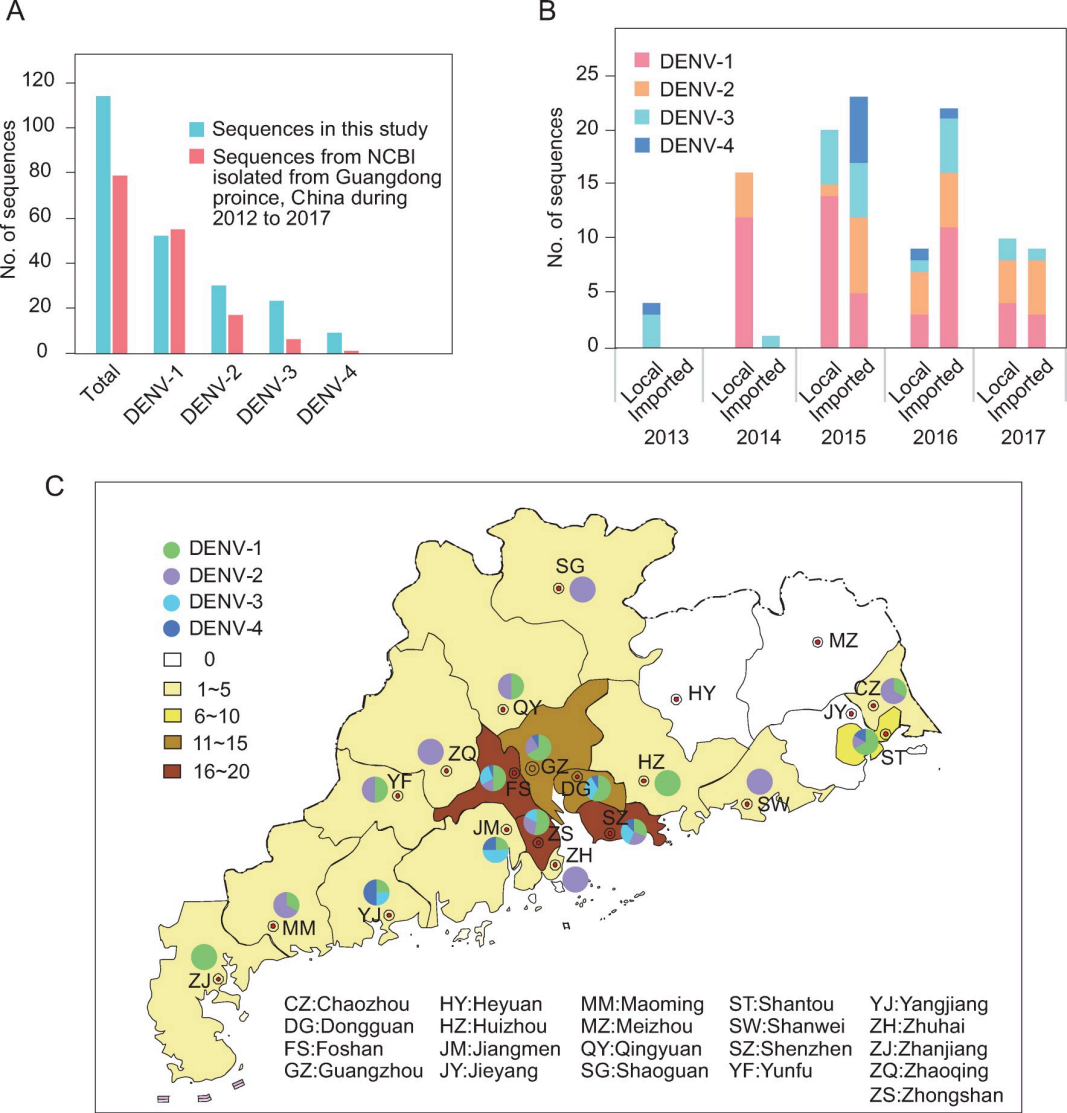
Statistical information of DENV genome sequences. (A) No. of DENV sequences both in the present study and those isolated from Guangdong province, China in GenBank from 2012 to 2017. (B, C) temporal distribution (B) and geographical distribution (C) of sequences in this study. “Local” means that sequences were isolated from human cases in Guangdong and “Imported” means that sequences were isolated from imported cases from other countries. The map image is copyright-free (available in standard map service system in China, http://bzdt.ch.mnr.gov.cn/).

One hundred and fourteen DENV complete genomes were sequenced in this study (DENV-1 = 52, DENV-2 = 30, DENV-3 = 23, DENV-4 = 9). In contrast, there were 79 published DENV genome sequences (DENV-1 = 55, DENV-2 = 17, DENV-3 = 6, DENV-4 = 1) isolated from Guangdong province, China from 2012 to 2017 in GenBank (Fig 1A). Except DENV-1, the sequence numbers of DENV2-4 in this study were more than those of published sequences (Fig 1A). In temporal distribution, there were 4, 17, 43, 31, and 19 DENV genome sequences from 2013 to 2017, respectively (Fig 1B). In 2015 and 2016, sequences from imported cases were more than sequences from local cases (23:20 in 2015, 22:9 in 2016) and in the other three years, it was the opposite (Fig 1B). Sequences from local cases were a little higher than sequences from imported cases (59:55), with ratio of 33:19 in DENV-1, 13:17 in DENV-2, 11:12 in DENV-3, 2:7 in DENV-4 (Fig 1B).

In geographical distribution, 75 of 114 DENV genome sequences were derived from five cities, Guangzhou, Shenzhen, Foshan, Dongguan, and Zhongshan (all five cities were located in the Pearl River Delta, Guangdong Province, which was characterized by developed economy, dense population, and frequent personnel flow). Moreover, sequences from Shenzhen and Foshan simultaneously included DENV1-4, and there were three DENV serotype sequences distributed in Guangzhou, Dongguan, Zhongshan, Shantou, Jiangmen, and Yangjiang. Only one DENV serotype was derived from Zhanjiang, Zhuhai, Zhaoqing, Shanwei, Huizhou, and Shaoguan. No sequences were distributed in Heyuan, Jieyang, or Meizhou (Fig 1C).

### Phylogenetic relationship

**Fig 2:**
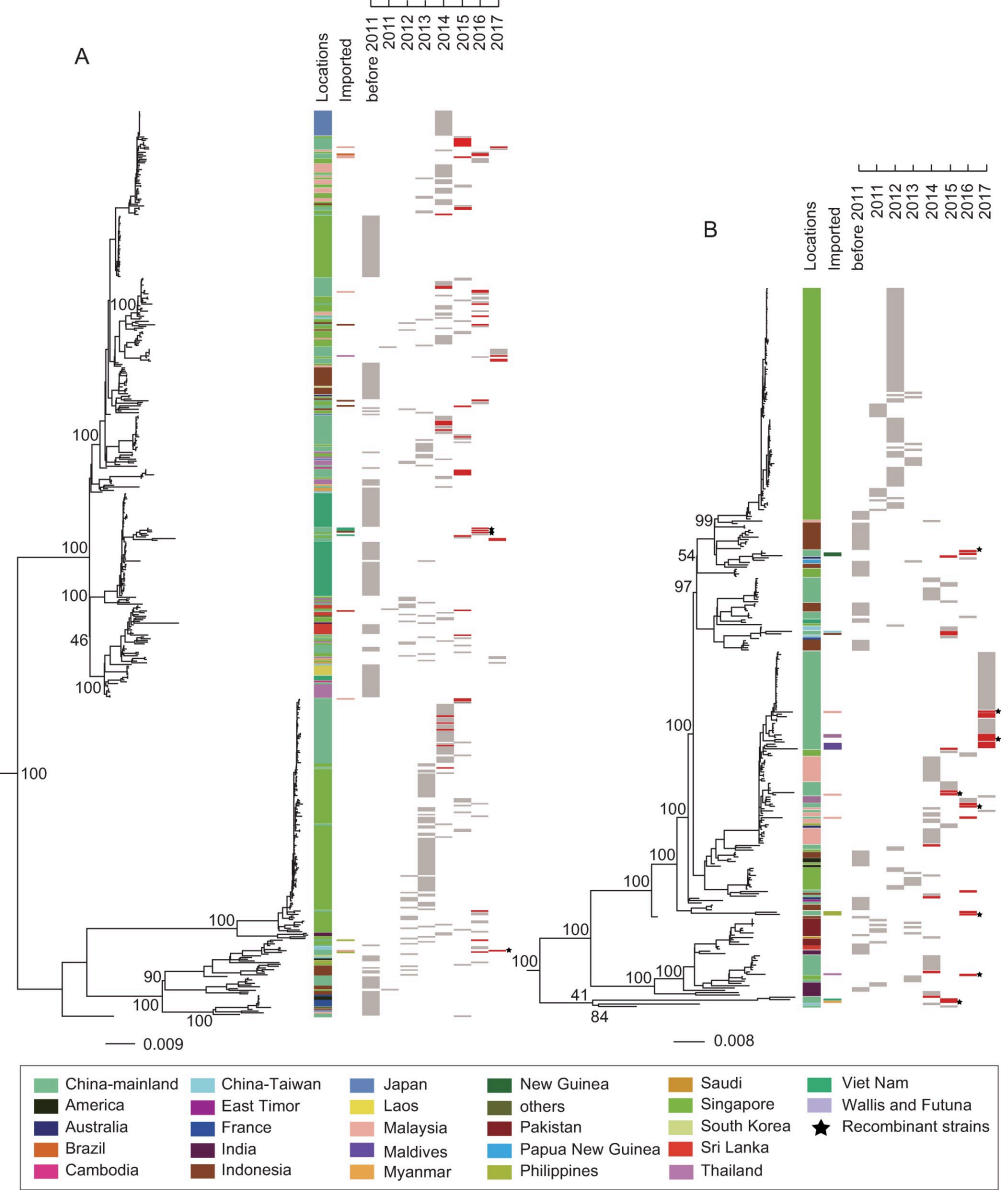
**Maximum-likelihood phylogenetic tree of DENV-1 (A) and DENV-2 (B) genomic coding regions.** UTR sequences and recombinant regions were excluded for phylogenetic analyses. Two published sequences (GenBank accession JQ920481, KX812530) were selected as their out-groups and the out-groups were removed for better presentation. “Locations” means the country where of sequences were obtained, and “Imported” means the original country of imported cases. Both are labelled by colored rectangles. The collection time are presented in red rectangles (sequences in this study) and in gray rectangles (sequences in GenBank). The recombinant isolates in this study are indicated by black stars. Bootstrap values are labelled at major nodes. Scale bar means nucleotide substitutions per site.

**Fig 3:**
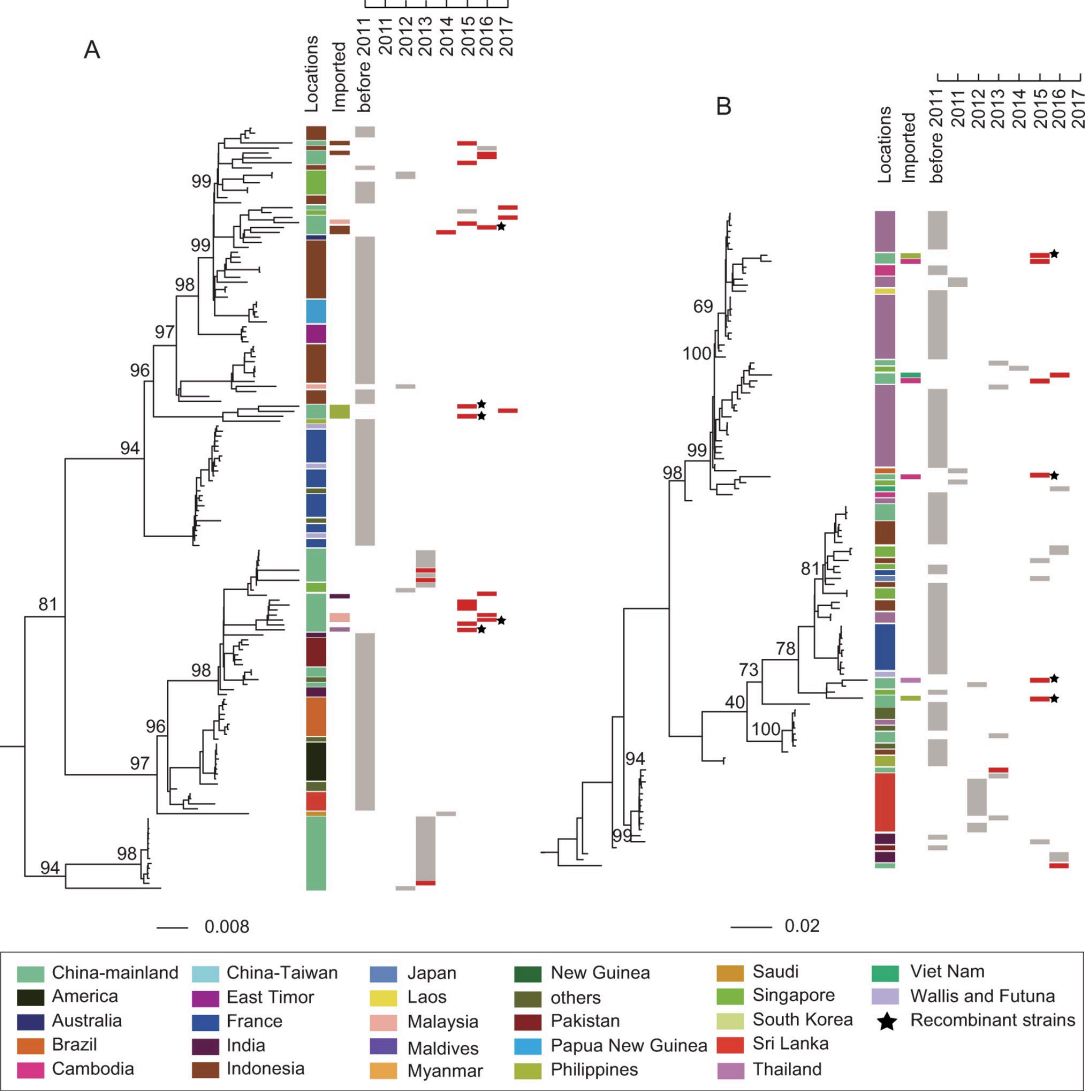
**Maximum-likelihood phylogenetic tree of DENV-3 (A) and DENV-4 (B) genomic coding regions.** Two published sequences (GenBank accession AB189121, AY037116) were selected as their out-groups and other description information are the same as in Fig 2.

To illuminate the phylogenetic relationship among DENV genome sequences in this study, we conducted maximum likelihood (ML) trees of DENV1-4. Our results showed that the sequences in this study presented a scattered distribution and they were not grouped into a single cluster in DENV1-4 ML trees (Figs 2 and 3). In addition, most imported cases of dengue infection were from Southeast and South Asian countries and most DENV isolates were closet to those from Southeast and South Asian countries (Figs 2 and 3). For DENV-1, the sequences from the same year showed a discrete distribution along ML tree and the sequences from different years were not grouped into a single cluster. Furthermore, the most imported cases of DENV-1 infection were from Malaysia, Indonesia, Thailand, Viet Nam, the Philippines, and Sri Lanka with only one case from Brazil. Most DENV-1 isolates in our study were closely related to isolates from Singapore, Malaysia, Indonesia, and Sri Lanka (Fig 2A). A similar distribution characteristic was observed in DENV-2 ML tree compared to DENV-1. The most imported DENV-2 cases were concentrated in Malaysia, Thailand, Maldives, the Philippines, Viet Nam, and Myanmar. Only a few imported DENV-2 cases were from Taiwan and New Guinea. Similarly, most DENV-2 isolates in our study were closely related to those from Malaysia, Singapore, and Indonesia (Fig 2B).

DENV3-4 sequences in this study were also characterized by a discrete distribution along ML trees (Fig 3). Meanwhile, most imported cases of DENV3-4 were from Malaysia, Indonesia, Thailand, India, the Philippines, Viet Nam, and Cambodia. Most isolates of DENV3-4 in the current study were closely related to those from Indonesia, Singapore, India, the Philippines, Thailand, and Cambodia (Fig 3).

Given that the potential recombinant events influence phylogenetic relationship, we further conducted ML trees of DENV1-4 without the recombinant regions excluded. The results showed that DENV1-4 sequences in the present study were not grouped into a single obvious cluster in their ML trees (S1 and S2 Figs) and most DENV isolates were closely related to those from Southeast and South Asian countries (S1 and S2 Figs). However, many isolates, especially the recombinant isolates (marked by black stars) changed their topological positions in the ML trees (Fig 2 and S1 Fig, Fig 3 and S2 Fig), which demonstrated that recombinant events took place in DENV genomes.

### Evidence of viral recombination

**Fig 4:**
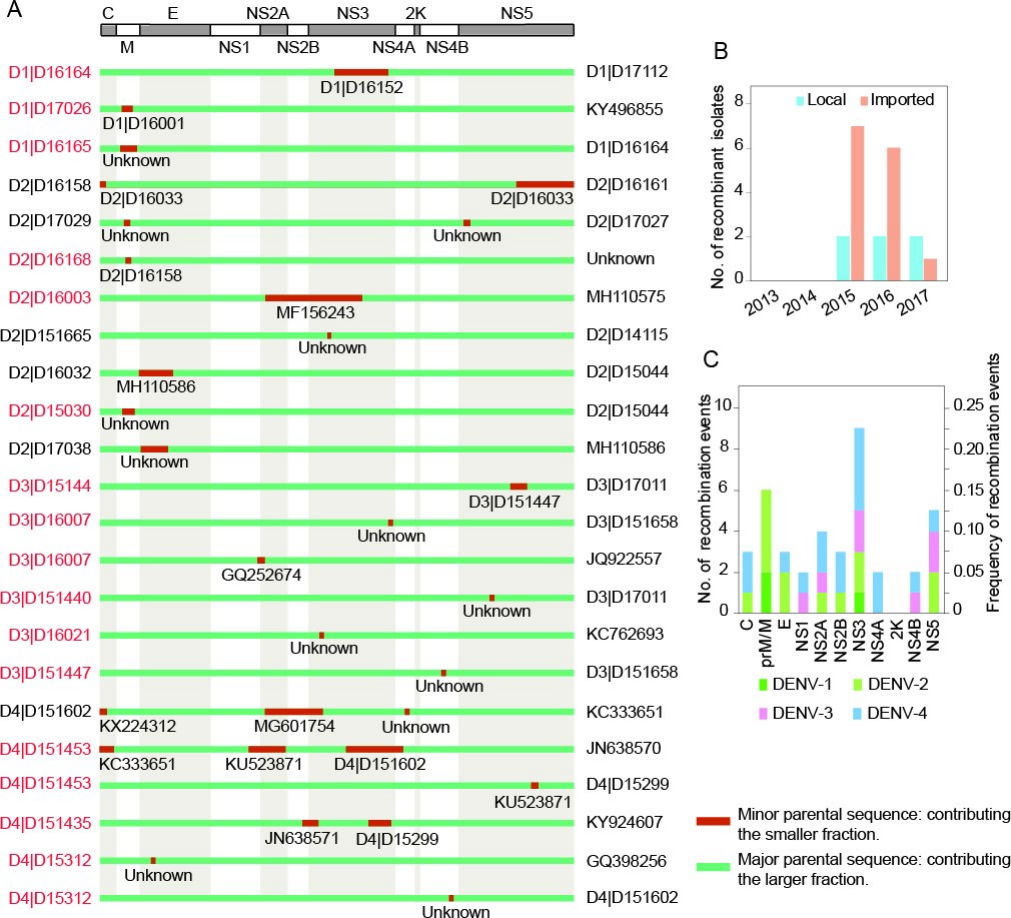
Recombinant information of DENV genomic coding regions. UTR sequences were excluded for recombinant analyses. (A) The recombinant region distribution along the viral genome. Because of the little difference in the CDS regions of DENV1-4, only one genomic organization of DENV is represented in the upper panel. In the lower panel, the recombinant isolates in this study are labelled on the left (with local isolates in black and imported isolates in red) and the major parental sequences of recombinant isolates are marked on the right, with minor parental sequences in the middle. D1, D2, D3, and D4 represent DENV-1, DENV-2, DENV-3, and DENV-4, respectively. “Unknown” means that only one parent sequence is detectable. (B) No. of recombinant isolates from Guangdong province, China (Local) or other countries (Imported) in indicated years. (C) Number (primary axis) and frequency (secondary axis) of recombinant event per DENV gene. Every involved gene will be counted once if the recombinant regions are across more than one gene, and the frequency per gene is calculated by its own recombination number divided by the total recombinant events.

Recombination analyses of DENV genome CDS regions was performed using Recombination Detection Program 4 (RDP4). Twenty intra-serotype recombinant isolates were identified in this study with three in DENV-1, eight in DENV-2, five in DENV-3, and four in DENV-4 (Fig 4A), and no inter-serotype recombinant events were detected. Among them, fourteen isolates were isolated from imported cases and six isolates were from local cases (Fig 4A). In temporal distribution, nine, eight, and three recombinant isolates were obtained in 2015, 2016, and 2017, respectively, but no recombinant isolates were obtained in 2013 or 2014 (Fig 4B).

The number and frequency of recombinant event in each DENV gene were further examined and the results showed that recombinant event occurred in every gene of DENV genome except *2K* (Fig 4A and 4C). The recombinant signal of *NS4A* was detected only in DENV-4, and other genes (but *2K*) were detected in at least two dengue serotypes. Furthermore, *NS3* not only had the most recombination events, but also the only one, in which recombination signal was detected simultaneously in DENV1-4. For the four DENV serotypes in this study, recombination signal was detected in two genes of DENV-1, seven genes of DENV-2, five genes of DENV-3, and nine genes of DENV-4 (Fig 4C). Meanwhile, DENV-1 had the highest sequences but the lowest recombination frequency (3/52) while DENV-4 had the highest recombination frequency (4/9).

### Nucleotide variation of dengue genome

**Fig 5:**
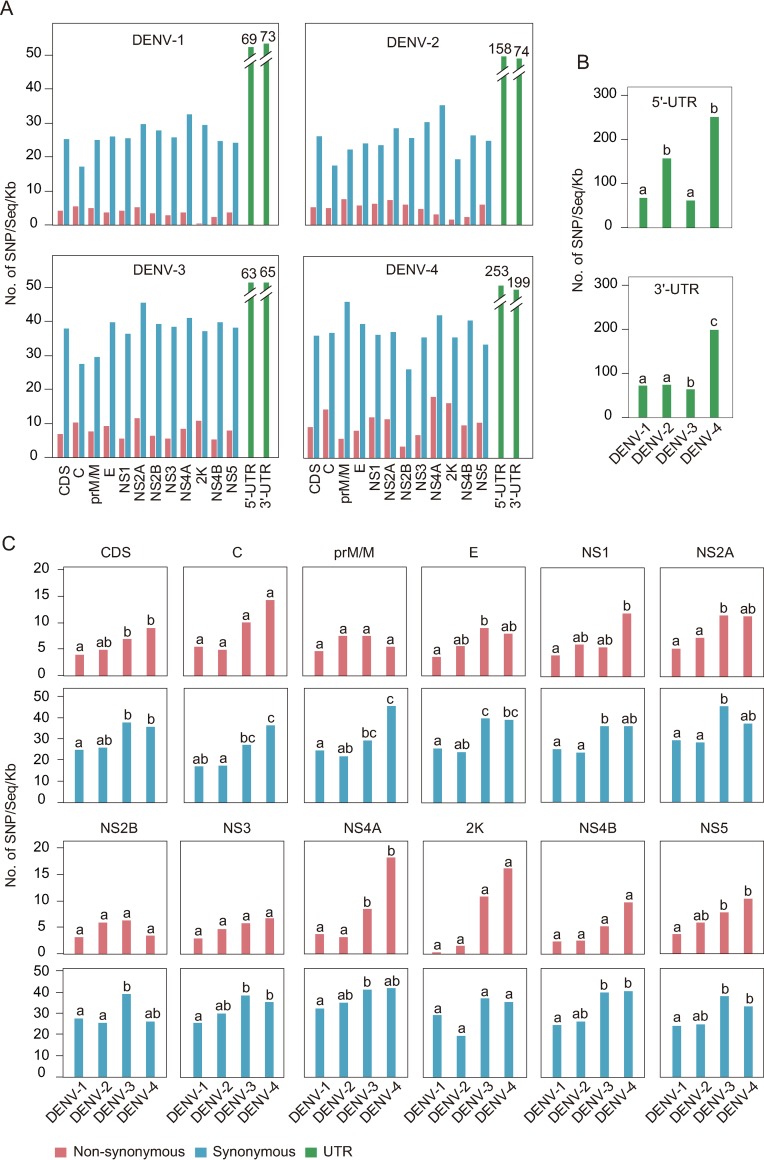
The normalized SNP distributions along DENV genome. (A) Intra-subtype comparison of normalized SNP distributions along DENV genomic regions. The normalized SNP are obtained by total SNPs per gene or UTR divided by the sequence number and gene length (kb). The normalized SNP of each gene is compared with that of CDS (one-tailed Fisher’s exact test for a 2 × 2 contingency table, and *p*-value < 0.01 is considered statistically significant). (B, C) Inter-subtype comparison of normalized SNP of UTR (B), non-synonymous mutations (C, red shade), and synonymous mutations (C, blue shade). Statistical significance was assessed using all pairwise Kruskal-Wallis one-way ANOVA test with *p* < 0.01. The values marked without the same superscript differ significantly.

We implemented single nucleotide polymorphism (SNP) analyses of dengue genome via homemade PERL script. Total synonymous (S) and non-synonymous (N) mutations in coding sequence (CDS) and SNP in UTR within each dengue subtype were calculated. The results showed that synonymous mutations were much higher than non-synonymous mutations or SNP in UTR in each serotype (S3A Fig). We further made a statistical analyses of SNP distribution along DENV genome within each subtype. The results showed that *NS5* had the maximum SNP while *2K* had the minimum (S3B Fig). Considering that both the sequence number in DENV1-4 and the sequence length of each gene varied, SNP was then normalized. The normalized SNP represented an even distribution along DENV genome and it was further compared with that of CDS to examine the variation of each gene within subtypes by a 2 × 2 contingency table. Our results showed that no significant difference was found in intra-serotype DENV genes (Fig 5A, *p* > 0.01), which indicated that each gene within the same serotype evolved at a similar rate.

Next, we performed inter-subtype comparison of each gene. For viral UTRs, 5'-UTR of DENV-2 and DENV-4 had more mutations than of DENV-1 and DENV-3. 3'-UTR of DENV-4 possessed the highest SNPs among the four subtypes (Fig 5B). In addition, rapider mutation of DENV3-4 than of DENV-1 occurred in both synonymous and non-synonymous SNP in CDS regions (Fig 5C, CDS panel, *p* < 0.01). Genetically, consistent inter-subtype differences occurred in three genes (*NS2A*, *2K*, and *NS5*) between synonymous and non-synonymous mutations (Fig 5C). For non-synonymous SNP, no significant inter-subtype difference was found in six genes (*C*, *M*, *NS2B*, *NS3*, *2K*, and *NS4B*, *p* > 0.01), and among the remaining five genes, *E* and *NS2A* of DENV-3 and *NS1*, *NS4A*, and *NS5* of DENV-4 exhibited the fastest variation rate, respectively. For synonymous SNP, significant inter-subtype differences occurred in all genes except *2K* (Fig 5C, *p* < 0.01). In general, more significant inter-subtype differences appeared in non-structural genes than in structural genes. Furthermore, normalized non-synonymous SNP were significantly different between DENV3-4 and DENV1-2, indicating that DENV3-4 evolved more rapidly than DENV1-2, especially DENV-4, which appeared to have the fastest mutation rate (Fig 5C).

## Discussion

In some Southeast Asia countries, Dengue fever outbreaks had been endemic [24, 25]. However, whether dengue disease is endemic in Guangdong province, China is controversial. Previous reports suggested that endemic prevalence introduced by imported isolates and endogenous epidemic outbreak co-triggered dengue outbreak in Guangdong in recent years [26–28], while Wang and his colleagues believed that DENV was an imported disease in Guangdong [29]. Compared to previous studies with conclusions made based on single *E* gene or few full-length genome sequences, the present study has implementation of phylogenetic, recombinant, and nucleotide variation analyses of 114 whole genome sequences combined with those published in GenBank of DENV1-4. This, we believe, could provide information that is much more reliable about molecular epidemiological characteristics of DENV in Guangdong in recent years.

Genomic sequencing has played a crucial role in the prevention and control of viral infectious disease [30, 31]. In this study, we sequenced 114 complete genomes of DENV1-4 from 2013 to 2017 and it provided a huge support for the research of dengue fever in Guangdong, China. In the meantime, DENV1-4 coexisted in Guangdong province, especially in the Pearl River Delta area, and this was in agreement with earlier reports [26]. Therefore, dengue epidemic situation in Guangdong is serious and further epidemiological surveillance on dengue disease is required.

To illuminate viral phylogenetic relationships, ML trees of DENV1-4 were conducted. As shown in Fig 2A and Fig 2B, sequences from Singapore (DENV-1 from 2012 to 2016 and DENV-2 from 2007 to 2013) were grouped into a single obvious cluster. It revealed that successive dengue outbreaks in Singapore were caused by endemic circulating strains. A similar characteristic was presented in sequences from Thailand (Fig 3B, DENV-4 from 1991 to 2010). All the above-mentioned characteristics showed that dengue fever had been endemic in Singapore and Thailand [32, 33]. However, DENV1-4 sequences from the same or different years in this study represented a scattered distribution along ML trees. Although four closely related DENV-3 sequences were successively isolated from 2014 to 2017, three of them were from imported cases outside Guangdong Province (Fig 3A). Furthermore, majority of isolates were closely related to those from Southeast and South Asian regions where most imported cases were obtained. This indicated that dengue epidemics in Guangdong were mainly induced by randomly imported cases from increasing tourism and economic exchanges between Southeast-South Asian countries and Guangdong province, China. Hence, we believe that dengue is still an imported disease in Guangdong.

Recombination is considered an important driver of viral evolution and adaption [34]. Numerous DENV recombinant events have been identified in previous studies [26, 27, 29, 34–37]. In this article, twenty recombinant isolates involving DENV1-4 were identified including each gene except *2K*, and this was further conformed by the inconsistency of ML trees constructed with or without recombinant regions. Frequent recombination generated multiple genotypes, giving rise to the current complicatedly epidemic situation in Guangdong (Figs 2 and 3). Among these recombinant isolates, fourteen isolates were from imported cases while only six isolates were from Guangdong. This means that the majority of recombinant events took place outside Guangdong, which indirectly indicated that dengue disease is not endemic in Guangdong Province. Among DENV genes, *NS3* possessed the most recombinant events covering simultaneously DENV1-4. This may be in connection with its function that plays a crucial role in viral replication [38]. The precise recombinant mechanism needs further investigation via co-infection model of different dengue strains *in vitro*.

Nucleotide mutation is another driver of viral evolution [39] and SNP analyses reflects the selection pressure within the viral population [40]. Our analyses showed that much more synonymous mutations were detected than non-synonymous mutations in DENV1-4, suggesting that most non-synonymous mutations of DENV1-4 were deleterious, and purification selection was shaping dengue virus population to prevent them from evolution *in situ*. Additionally, there were no obvious intra-serotype differences in DENV genes, but more significant inter-subtype differences in non-structural genes than in structural genes were detected. This was possibly because of the biological functional difference between the structural and non-structural proteins. For flavivirus, the structural proteins constitute the virion and they mediate cellular adsorption, penetration, and fusion while the non-structural proteins combined with their UTRs participate in viral replication, translation, and impairment of host antiviral responses [41–44]. The specific molecular mechanism still needs further investigation.

Recently, the first vaccine for the prevention of dengue disease has been approved by the U.S. Food and Drug Administration, but there are some limitations in its global inoculation (https://www.fda.gov/news-events/press-announcements/first-fda-approved-vaccine-prevention-dengue-disease-endemic-regions). Moreover, the humid and rainy climate in Guangdong is suitable for the multiplication of mosquitoes and DENV transmission. Therefore, measures that are more effective for dengue prevention and control are urgently needed in the future. Our investigation has provided further knowledge on the prevalence of DENV in Guangdong and it is in favor with the formulation of prevention and control strategies for dengue infection.

## Supporting information

S1 Checklist STROBE checklist. (PDF)

S1 Fig **Maximum-likelihood phylogenetic tree of DENV-1 (A) and DENV-2 (B) genome coding regions** (The recombinant regions of DENV1-2 sequences are not excluded and other information are described in Fig 2).
(TIF)

S2 Fig **Maximum-likelihood phylogenetic tree of DENV-3 (A) and DENV-4 (B) genome coding regions** (The recombinant regions of DENV3-4 sequences are not excluded and other information are described in Fig 3).
(TIF)

S3 Fig The total SNPs among DENV genome. (A) Total SNPs within UTR (green), non-synonymous (red) and synonymous (blue) mutations of DENV1-4. (B) Total SNPs among each gene or UTR of DENV1-4.
(TIF)

S1 Table The PCR primer sequences used in this study. (PDF)

S2 Table GenBank accessions of published DENV sequences in dataset. (PDF)

S3 Table Recombination test information. (PDF)

S4 Table GenBank accessions of DENV sequences in this study. (PDF)
