# Supplementary material for: Genomic epidemiological characteristics of dengue fever in Guangdong province, China from 2013 to 2017
Source: PLoS Negl Trop Dis. 2020 Mar 3;14(3):e0008049. doi: 10.1371/journal.pntd.0008049 (PMC7053713; doi:10.1371/journal.pntd.0008049)
Supplement: S1 Checklist — (PDF) [file pntd.0008049.s001.pdf]

STROBE Statement—checklist of items that should be included in reports of observational studies

	Item No	Recommendation
**Title and abstract**	1	(*a*) Title
		(*b*) Abstract and author summary
**Introduction**		
Background/rationale	2	Introduction, paragraph 1-3
Objectives	3	Introduction, paragraph 4
**Methods**		
Study design	4	Methods, paragraph 1-5
Setting	5	
Participants	6	
Variables	7	
Data sources/ measurement	8*	Methods, paragraph 2-5
Bias	9	
Study size	10	
Quantitative variables	11	
Statistical methods	12	Fig 5 legend

**Results**		
Participants	13*	
Descriptive data	14*	Results, paragragh 1-2
Outcome data	15*	Results, paragragh 3-9
Main results	16	Results, paragragh 1-9
Other analyses	17	
**Discussion**		
Key results	18	Discussion, paragragh 1-4
Limitations	19	Discussion, paragragh 4
Interpretation	20	Discussion, paragragh 1-6
Generalisability	21	Discussion, paragragh 1-6
**Other information**		
Funding	22	

Note: An Explanation and Elaboration article discusses each checklist item and gives methodological background and published examples of transparent reporting. The STROBE checklist is best used in conjunction with this article (freely available on the Web sites of PLoS Medicine at http://www.plosmedicine.org/, Annals of Internal Medicine at http://www.annals.org/, and Epidemiology at http://www.epidem.com/). Information on the STROBE Initiative is available at www.strobe-statement.org.
