# Supplementary figures and images for: Genomic epidemiological characteristics of dengue fever in Guangdong province, China from 2013 to 2017
Source: PLoS Negl Trop Dis. 2020 Mar 3;14(3):e0008049. doi: 10.1371/journal.pntd.0008049 (PMC7053713; doi:10.1371/journal.pntd.0008049)

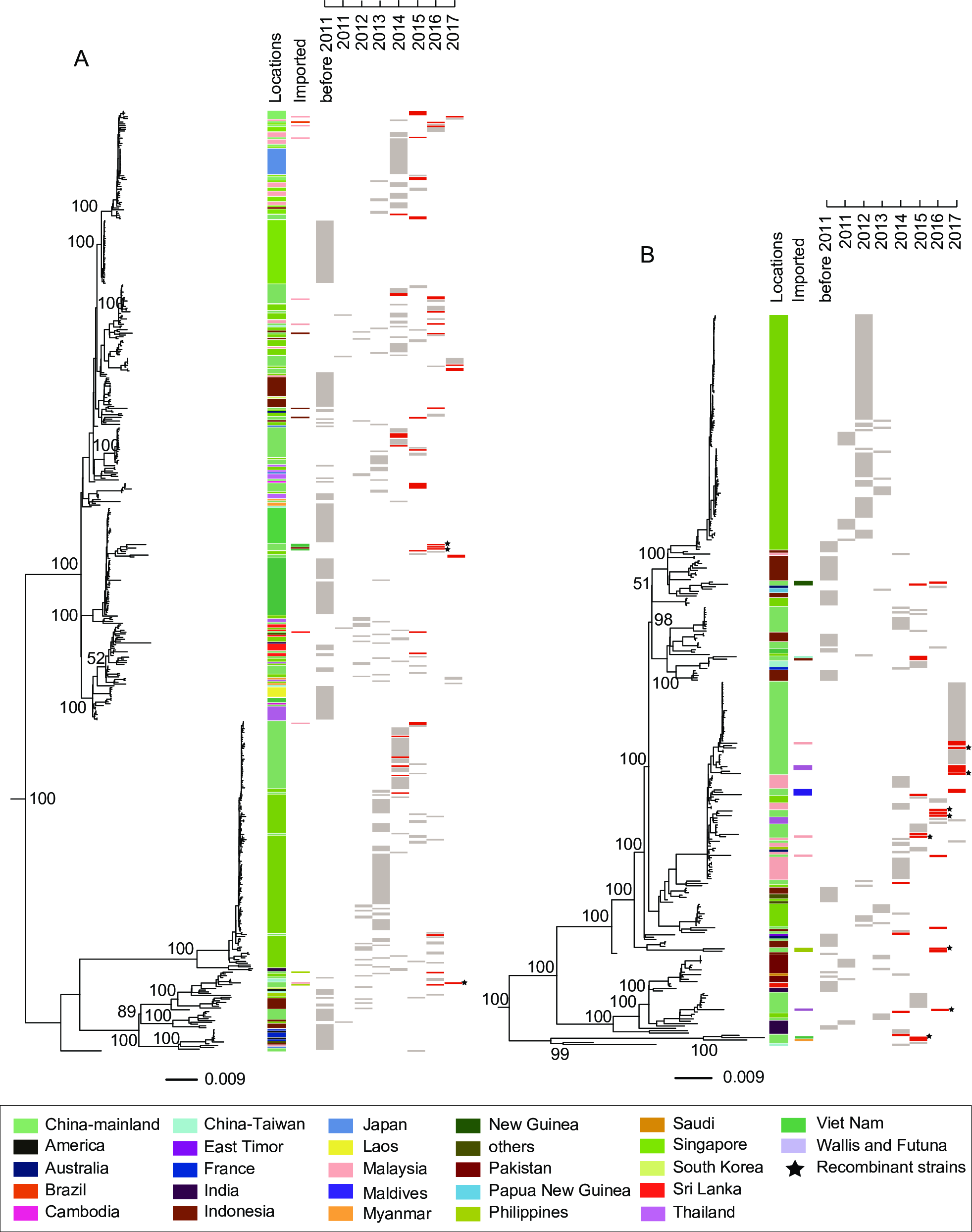

Supplement: S1 Fig — Maximum-likelihood phylogenetic tree of DENV-1 (A) and DENV-2 (B) genome coding regions (The recombinant regions of DENV1-2 sequences are not excluded and other information are described in Fig 2). (TIF) [file pntd.0008049.s002.tif]

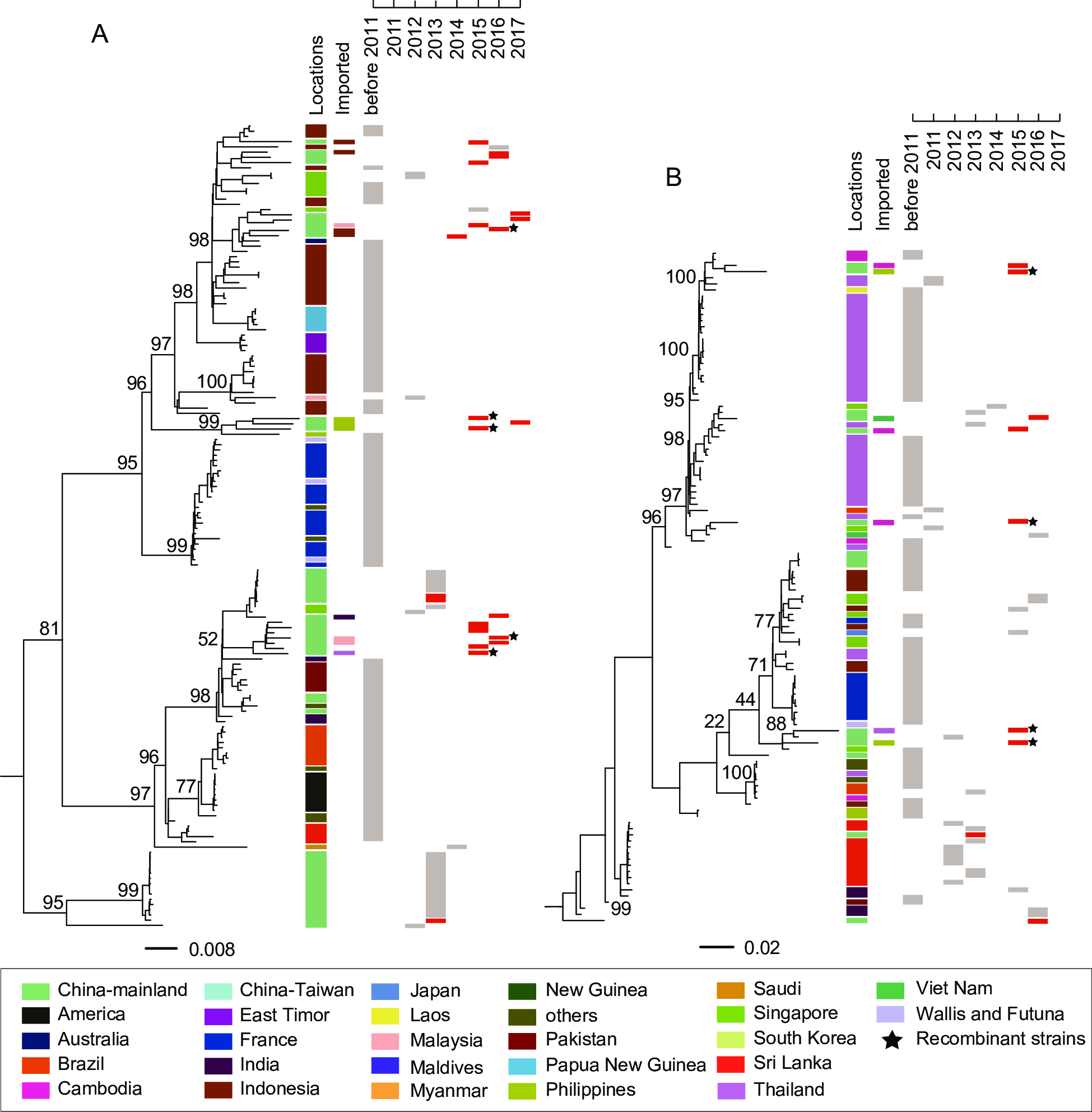

Supplement: S2 Fig — Maximum-likelihood phylogenetic tree of DENV-3 (A) and DENV-4 (B) genome coding regions (The recombinant regions of DENV3-4 sequences are not excluded and other information are described in Fig 3). (TIF) [file pntd.0008049.s003.tif]

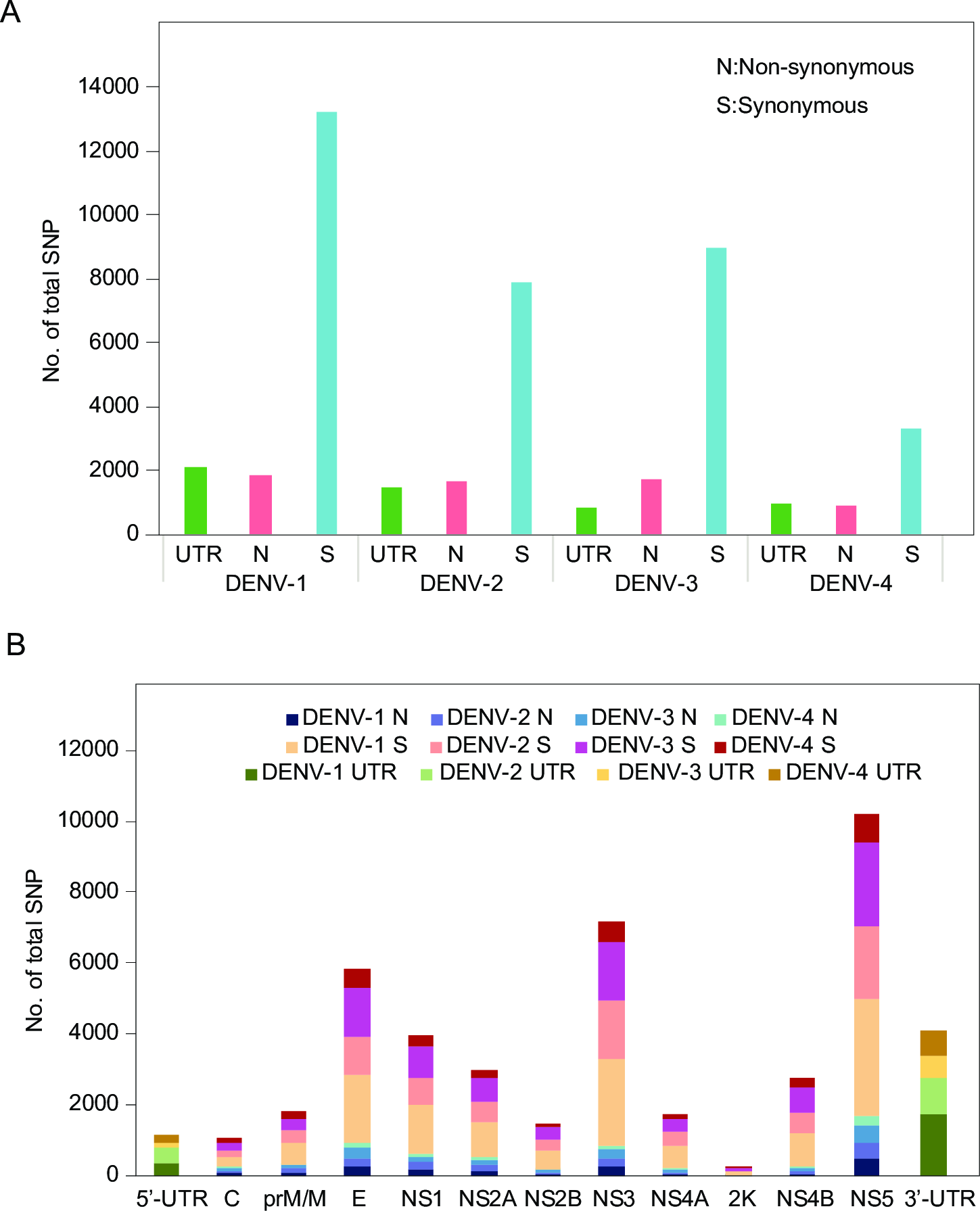

Supplement: S3 Fig — (A) Total SNPs within UTR (green), non-synonymous (red) and synonymous (blue) mutations of DENV1-4. (B) Total SNPs among each gene or UTR of DENV1-4. (TIF) [file pntd.0008049.s004.tif]
