# Supplementary material for: Genomic epidemiological characteristics of dengue fever in Guangdong province, China from 2013 to 2017
Source: PLoS Negl Trop Dis. 2020 Mar 3;14(3):e0008049. doi: 10.1371/journal.pntd.0008049 (PMC7053713; doi:10.1371/journal.pntd.0008049)
Supplement: S1 Table — (PDF) [file pntd.0008049.s005.pdf]

S1 Table. PCR primer sequence

Primer	Sequence (5'-3')
D1F1	1-AGTAGTTAGTCTACGTGGACCGA-23
D1R1	1718-TGCATTGCTCCTTCTTGTGATCCTA-1692
D1F2	1581-TACCACTGCCTTGGACCTC-1599
D1R2	3338-TCTTTCCTGTGACTGTTGTGGT-3317
D1F3	3275-GTTGTGGATGAACATTGTGGAAATC-3299
D1R3	4681-CTGGTGACRTGCCACATTGT-4661
D1F4	4559-GAAAGAGCAGTYCTTGATGATGG-4581
D1R4	6264-ATCTCCACGTCCATGTTCTCCT-6243
D1F5	6171-TTGCCTCAGAAGGCTTCCA-6189
D1R5	7866-TGGTGACTTTCTTCAGCCCAGCGCA-7844
D1F6	7803-TCATAGACCTCGGTTGTGGAAGA-7825
D1R6	9252-TGTTCRGGTTCCATGATGTCAGT-9230
D1F7	9158-ATGTATGCAGATGAYACAGCCGGAT-9182
D1R7	10735-AGAACCTGTTGATTCAACMGCA-10714
D2F1	1-AGTWGTTAGTCTACGTGGACCGA-23
D2R1	1724-CTCYTTCTGTATCCAATTTGATCCTTGT-1740
D2F2	1577-GGCTAYGGCACTGTCACGATGGA-1598
D2R2	3306-TCCTCTATTTCCACRGTCCTCAGTCA-3281
D2F3	3178-CARCACAACTAYAGACCAGGCTA-3200
D2R3	4661-TGGAATGTTCCTTCTTTRTAAACTCCA-4635
D2F4	4563-AAAGGCBGAACTGGAAGATGGAG-4586
D2R4	6025-TCRAACATACTGGGAATGATTCCTTC-6047
D2F5	ATGATGAAGACTGCGCACACTGGA
D2R5	CTTTCCCTTCTGGTGTGACAAGGTT
D2F6	7645-CAGATCTACAAGAAAAGTGGAATCCA-7673
D2R6	9252-GTGTTCTCCTTCCATGTGGTTTGT-9229
D2F7	9175-GCAGGATGGGACACAAGAAT-9194
D2R7	10700-GCAGAACCTGTTGATTCAACAGCACCA-10724
D3F1	1-AGTTGTTAGTCTACGTGGACCGA-23
D3R1	1707-ATTGCTCCCTCTTGCGATCCAA-1686
D3F2	1526-ACAATGAAGAACAAAGCATGGATGGT-1551
D3R2	3235-CTTCACAATAGTTGAAGTCCAGCTC-3259
D3F3	3129-GAGTGACATGATYATYCCAAAGAGTCT-3155
D3R3	4663-CCTCTTGTRACGTGCCACAT-4682
D3F4	4574-TTCCACACCATGTGGCACGT-4593
D3R4	6308-ATCTGAATAAGTGCGGGCATCAAGC-6322
D3F5	6122-AACTCATGAGGAGGGGTGACCT- 6141
D3R5	7689-CCTCTTTTYAACCCTTCTTTGGCTTC-7715
D3F6	7498-AGGGAGCTATYTAGCAGGAGCTG-7519
D3R6	9180-GTCATCTTCTGTTATTCTTGTGTCCCA-9206
D3F7	9180-ATACCYGGAGGAGCCATGTATGC-9173
D3R7	10707-AGAACCTGTTGATTCAACAGCACCA-10683
D4F1	1-AGTTGTTAGTCTGTGTGGACCGA-23
D4R1	1668-AGCACTGTCACATCCTGTCTCTT-1688
D4F2	1630-GGAATYACAAAGARAGAATGGTGAC-1655
D4R2	3473-ACCTGTGATTTRACCATGTTCT-3452
D4F3	3319-GACCACCACTGCATCTGGAAA-3339

Table S1 PCR primer sequence

Primer	Sequence (5'-3')
D4R3	4748-ATCCCCCACCGTATGATATCATGT-4771
D4F4	4724-GTRTTTCAYACAATGTGGCATGT-4745
D4R4	6351-CACTGGCAAACTCCTTGAAATCCTT-6375
D4F5	6250-AAGAAAACATGGAGGTTGAAATTTGGACTA-6279
D4R5	7837-ACGTTCTTGAGTGTBGCCATGTA-7859
D4F6	7738-AGATGGATYGTTGARAGAGGGATGGT-7763
D4R6	9304-TCGGTGTGGGTCTGAGGACTTT-9325
D4F7	9256-TAGCCAAAGCCATTTTCAAACTAAC-9278
D4R7	10670-AGAACCTGTTGGATCAACAACAC-10648
