# Supplementary material for: Genomic epidemiological characteristics of dengue fever in Guangdong province, China from 2013 to 2017
Source: PLoS Negl Trop Dis. 2020 Mar 3;14(3):e0008049. doi: 10.1371/journal.pntd.0008049 (PMC7053713; doi:10.1371/journal.pntd.0008049)
Supplement: S2 Table — (PDF) [file pntd.0008049.s006.pdf]

S2 Table. GenBank accession of published DENV sequences

| DENV-1 | DENV-2 | DENV-3 | DENV-4 |
|---|---|---|---|
| AB178040 | AB189122 | AB189125 | AF326573 |
| AB189120 | AB189123 | AB189126 | AY618990 |
| AB189121 | AB189124 | AB189127 | AY618991 |
| AB195673 | AY037116 | AB189128 | AY618992 |
| AB608787 | DQ448231 | AB214879 | AY618993 |
| AY708047 | EU081177 | AB214880 | AY858050 |
| AY713476 | EU081178 | AB214881 | FJ196849 |
| AY726554 | EU081179 | AB214882 | GQ398256 |
| AY732479 | EU081180 | AY099337 | GQ868594 |
| AY732482 | EU179857 | AY744677 | GU289913 |
| AY835999 | EU179858 | AY744678 | HQ875339 |
| DQ193572 | EU482640 | AY744679 | JF741967 |
| DQ672556 | EU482672 | AY744680 | JN559740 |
| DQ672557 | FJ196853 | AY744681 | JN638570 |
| DQ672558 | FJ898454 | AY744682 | JN638571 |
| DQ672559 | GQ252676 | AY744683 | JQ513345 |
| DQ672560 | GQ252677 | AY744684 | JQ822247 |
| DQ672561 | GQ398264 | AY744685 | JQ915081 |
| DQ672563 | GQ398265 | AY770511 | JQ915082 |
| DQ672564 | GQ398266 | AY858037 | JQ915083 |
| EF032590 | GQ398267 | AY858038 | JQ915084 |
| EU081226 | HM488257 | AY858039 | JQ915085 |
| EU081227 | JF327392 | AY858040 | JQ915086 |
| EU081228 | JQ922549 | AY858041 | JQ915087 |
| EU081229 | JQ955624 | AY858042 | JQ915088 |
| EU081230 | JX470186 | AY858043 | JQ915089 |
| EU081231 | KC131142 | AY858044 | JQ915090 |
| EU081232 | KC762655 | AY858045 | JQ922560 |
| EU081233 | KC762656 | AY858046 | JX024757 |
| EU081234 | KC762657 | AY858047 | JX024758 |
| EU081236 | KC762658 | AY858048 | KC333651 |
| EU081238 | KC762659 | EU081221 | KC762694 |
| EU081240 | KC762660 | EU081223 | KC762695 |
| EU081242 | KC762661 | EU482558 | KC762696 |
| EU081244 | KC762662 | EU482559 | KC762697 |
| EU081245 | KC762663 | EU482596 | KC762698 |
| EU081246 | KC762664 | EU687218 | KC762699 |
| EU081247 | KC762665 | EU726771 | KF041260 |
| EU081248 | KC762666 | FJ182013 | KF907503 |
| EU081249 | KC762667 | FJ390376 | KF955510 |

| | | | |
|---|---|---|---|
| EU081250 | KC762668 | FJ547070 | KM190936 |
| EU081252 | KC762669 | FJ644564 | KP723482 |
| EU081253 | KC762670 | FJ882571 | KP792537 |
| EU081259 | KC762671 | FJ882575 | KR011349 |
| EU081261 | KC762672 | FJ882576 | KT026308 |
| EU081263 | KC762673 | FJ898455 | KT026310 |
| EU081264 | KC762674 | FJ898456 | KU513442 |
| EU081265 | KC762675 | GQ252674 | KU523872 |
| EU081266 | KC762676 | GU363549 | KX059018 |
| EU081267 | KC762677 | GU370052 | KX059020 |
| EU081268 | KC762678 | JF504679 | KX059023 |
| EU081269 | KC762679 | JN406515 | KX059026 |
| EU081270 | KC762680 | JN662391 | KX059027 |
| EU081271 | KF041232 | JQ411814 | KX059031 |
| EU081273 | KF041233 | JQ920476 | KX059032 |
| EU081276 | KF041234 | JQ920477 | KX059034 |
| EU081277 | KF041235 | JQ920478 | KX059035 |
| EU081279 | KF041237 | JQ920479 | KX059036 |
| EU081280 | KF360005 | JQ920480 | KX224312 |
| EU359008 | KF479233 | JQ920481 | KX812530 |
| EU482483 | KJ010185 | JQ920482 | KX845005 |
| EU482489 | KJ010186 | JQ920483 | KY451945 |
| EU482493 | KJ701507 | JQ920484 | KY586827 |
| EU482498 | KJ734727 | JQ920485 | KY586828 |
| EU482513 | KJ830750 | JQ920486 | KY586829 |
| EU482517 | KM217156 | JQ920487 | KY586830 |
| EU482708 | KM217158 | JQ920488 | KY586831 |
| EU482709 | KM279515 | JQ920489 | KY586832 |
| EU482716 | KM279517 | JX669489 | KY586833 |
| EU482718 | KM279518 | JX669490 | KY586834 |
| EU482801 | KM279519 | JX669491 | KY586835 |
| EU482811 | KM279520 | JX669492 | KY586836 |
| EU660397 | KM279521 | JX669493 | KY586837 |
| EU677154 | KM279522 | JX669495 | KY586838 |
| EU677161 | KM279523 | JX669497 | KY586839 |
| EU677171 | KM279524 | JX669499 | KY586898 |
| EU677172 | KM279525 | KC261634 | KY586903 |
| EU863650 | KM279526 | KC762681 | KY586904 |
| FJ024426 | KM279528 | KC762682 | KY586908 |
| FJ024428 | KM279529 | KC762683 | KY586909 |
| FJ024429 | KM279530 | KC762684 | KY586910 |
| FJ024435 | KM279531 | KC762685 | KY586912 |
| FJ024437 | KM279532 | KC762686 | KY586914 |

| | | | |
|---|---|---|---|
| FJ024443 | KM279533 | KC762687 | KY586917 |
| FJ024448 | KM279534 | KC762688 | KY586918 |
| FJ024453 | KM279535 | KC762689 | KY586919 |
| FJ024455 | KM279536 | KC762690 | KY586922 |
| FJ024457 | KM279537 | KC762691 | KY586932 |
| FJ024472 | KM279538 | KC762692 | KY586935 |
| FJ176779 | KM279539 | KC762693 | KY586938 |
| FJ182022 | KM279540 | KF041254 | KY586939 |
| FJ182026 | KM279541 | KF041255 | KY586940 |
| FJ182027 | KM279542 | KF041256 | KY849762 |
| FJ182028 | KM279543 | KF041257 | KY921909 |
| FJ182029 | KM279544 | KF041258 | KY921910 |
| FJ182036 | KM279545 | KF041259 | KY924607 |
| FJ196841 | KM279546 | KF824902 | LC069810 |
| FJ196842 | KM279547 | KF824903 | MF004387 |
| FJ196844 | KM279548 | KF954945 | MG272272 |
| FJ196845 | KM279549 | KF954946 | MG272274 |
| FJ205881 | KM279550 | KF954947 | MG601754 |
| FJ390386 | KM279551 | KF954948 | |
| FJ390388 | KM279552 | KF954949 | |
| FJ410205 | KM279553 | KF955474 | |
| FJ410236 | KM279554 | KJ622191 | |
| FJ410239 | KM279555 | KJ622192 | |
| FJ410261 | KM279556 | KJ622193 | |
| FJ410276 | KM279557 | KJ622194 | |
| FJ410283 | KM279558 | KJ622195 | |
| FJ410286 | KM279559 | KJ622196 | |
| FJ410289 | KM279560 | KJ622197 | |
| FJ432719 | KM279561 | KJ622198 | |
| FJ461303 | KM279562 | KJ622199 | |
| FJ461315 | KM279563 | KJ830751 | |
| FJ461328 | KM279564 | KR296743 | |
| FJ639669 | KM279565 | KR296744 | |
| FJ687426 | KM279566 | KU509279 | |
| FJ687427 | KM279567 | KU509281 | |
| FJ687428 | KM279568 | KU509282 | |
| FJ687429 | KM279569 | KX380839 | |
| FJ687430 | KM279570 | KX380840 | |
| FJ687431 | KM279571 | KX380841 | |
| FJ687432 | KM279572 | KX380842 | |
| FJ687433 | KM279573 | KY794786 | |
| FJ850068 | KM279574 | KY794787 | |
| FJ906963 | KM279575 | KY794788 | |

| | | | |
|---|---|---|---|
| GQ199794 | KM279576 | KY794789 | |
| GQ398255 | KM279577 | KY794790 | |
| GQ868602 | KM279578 | KY863456 | |
| GQ868611 | KM279579 | KY921906 | |
| GU131732 | KM279580 | MF004386 | |
| GU131739 | KM279581 | | |
| HM181952 | KM279582 | | |
| HM469968 | KM279587 | | |
| HQ891313 | KM279588 | | |
| HQ891314 | KM279589 | | |
| HQ891315 | KM279590 | | |
| HQ891316 | KM279591 | | |
| JN054255 | KM279592 | | |
| JN054256 | KM279593 | | |
| JN205310 | KM279594 | | |
| JN544411 | KM279595 | | |
| JN697056 | KM279596 | | |
| JN697057 | KM279597 | | |
| JQ048541 | KM279598 | | |
| KC759167 | KM279599 | | |
| KC762620 | KM279600 | | |
| KC762623 | KM279601 | | |
| KC762625 | KP012546 | | |
| KC762628 | KP723478 | | |
| KC762630 | KP723479 | | |
| KC762631 | KR779782 | | |
| KC762633 | KR779786 | | |
| KC762634 | KT187553 | | |
| KC762635 | KT187554 | | |
| KC762636 | KT187555 | | |
| KC762637 | KT187556 | | |
| KC762638 | KT187557 | | |
| KC762640 | KT187558 | | |
| KC762641 | KU094070 | | |
| KC762642 | KU365901 | | |
| KC762644 | KU365902 | | |
| KC762648 | KU365903 | | |
| KC762649 | KU509271 | | |
| KC762651 | KU517845 | | |
| KC762652 | KU517846 | | |
| KC762653 | KU517847 | | |
| KC762654 | KU948303 | | |
| KF887994 | KX225485 | | |

| | | | |
|---|---|---|---|
| KF971869 | KX225486 | | |
| KF971870 | KX372564 | | |
| KF971871 | KX380807 | | |
| KJ438293 | KX380808 | | |
| KJ438296 | KX380809 | | |
| KJ468234 | KX380810 | | |
| KJ726662 | KX380811 | | |
| KJ726663 | KX380812 | | |
| KJ726664 | KX380813 | | |
| KJ755855 | KX380814 | | |
| KJ806939 | KX380816 | | |
| KJ806941 | KX380817 | | |
| KJ806943 | KX380819 | | |
| KJ806944 | KX380820 | | |
| KJ806945 | KX380822 | | |
| KJ806946 | KX380823 | | |
| KJ806947 | KX380824 | | |
| KJ806949 | KX380825 | | |
| KJ806950 | KX380826 | | |
| KJ806951 | KX380827 | | |
| KJ806953 | KX380828 | | |
| KJ806959 | KX380829 | | |
| KJ806961 | KX380830 | | |
| KJ806963 | KX380831 | | |
| KJ933413 | KX380832 | | |
| KM403575 | KX380833 | | |
| KM403576 | KX380834 | | |
| KM403577 | KX380835 | | |
| KM403578 | KX380837 | | |
| KM403579 | KX380838 | | |
| KM403580 | KX452015 | | |
| KM403581 | KX452016 | | |
| KM403582 | KX452017 | | |
| KM403583 | KX452018 | | |
| KM403584 | KX452019 | | |
| KM403585 | KX452020 | | |
| KM403586 | KX452021 | | |
| KM403587 | KX452022 | | |
| KM403588 | KX452024 | | |
| KM403589 | KX452025 | | |
| KM403590 | KX452026 | | |
| KM403591 | KX452027 | | |
| KM403592 | KX452028 | | |

| | | | |
|---|---|---|---|
| KM403593 | KX452029 | | |
| KM403594 | KX452030 | | |
| KM403595 | KX452031 | | |
| KM403596 | KX452032 | | |
| KM403597 | KX452033 | | |
| KM403598 | KX452037 | | |
| KM403599 | KX452038 | | |
| KM403600 | KX452039 | | |
| KM403601 | KX452040 | | |
| KM403602 | KX452045 | | |
| KM403603 | KX452046 | | |
| KM403604 | KX621245 | | |
| KM403605 | KX621246 | | |
| KM403606 | KX621247 | | |
| KM403607 | KX621248 | | |
| KM403608 | KX655788 | | |
| KM403609 | KY427085 | | |
| KM403610 | KY794785 | | |
| KM403611 | KY921904 | | |
| KM403612 | KY921905 | | |
| KM403613 | KY923048 | | |
| KM403614 | LC111438 | | |
| KM403615 | LC410189 | | |
| KM403616 | LC410190 | | |
| KM403617 | LC410191 | | |
| KM403618 | MF004385 | | |
| KM403619 | MF043956 | | |
| KM403620 | MF156236 | | |
| KM403621 | MF156237 | | |
| KM403622 | MF156238 | | |
| KM403623 | MF156239 | | |
| KM403624 | MF156240 | | |
| KM403625 | MF156241 | | |
| KM403626 | MF156247 | | |
| KM403627 | MF314189 | | |
| KM403628 | MG560143 | | |
| KM403629 | MG560144 | | |
| KM403630 | MH110564 | | |
| KM403631 | MH110565 | | |
| KM403632 | MH110566 | | |
| KM403633 | MH110567 | | |
| KM403634 | MH110568 | | |
| KM403635 | MH110569 | | |

| | | | |
|---|---|---|---|
| KM403636 | MH110570 | | |
| KP398852 | MH110571 | | |
| KP406802 | MH110572 | | |
| KP406803 | MH110573 | | |
| KP686070 | MH110574 | | |
| KP723473 | MH110576 | | |
| KP723476 | MH110577 | | |
| KP772252 | MH110578 | | |
| KR024705 | MH110579 | | |
| KR024706 | MH110580 | | |
| KR024707 | MH110581 | | |
| KR024708 | MH110582 | | |
| KR028435 | MH110586 | | |
| KR052012 | MH110587 | | |
| KR071622 | MH110588 | | |
| KR919820 | MH110590 | | |
| KR919821 | MH110591 | | |
| KT187559 | MH110593 | | |
| KT187560 | MH110594 | | |
| KT187561 | MH110595 | | |
| KT187562 | MH110596 | | |
| KT187563 | MH110597 | | |
| KT187564 | MH110598 | | |
| KT827364 | MH110599 | | |
| KT827365 | MH110600 | | |
| KT827366 | MH110601 | | |
| KT827367 | MH110602 | | |
| KT827370 | MH110603 | | |
| KT827371 | MH985858 | | |
| KT827372 | | | |
| KT827373 | | | |
| KT827374 | | | |
| KT827375 | | | |
| KT827376 | | | |
| KT827377 | | | |
| KT827378 | | | |
| KT827379 | | | |
| KT831765 | | | |
| KU094071 | | | |
| KU365900 | | | |
| KU509250 | | | |
| KU509257 | | | |
| KU509263 | | | |

| | | | |
|---|---|---|---|
| KU509266 | | | |
| KU666939 | | | |
| KU666940 | | | |
| KU666941 | | | |
| KU666942 | | | |
| KX224261 | | | |
| KX224263 | | | |
| KX225483 | | | |
| KX225484 | | | |
| KX225488 | | | |
| KX225489 | | | |
| KX225490 | | | |
| KX225491 | | | |
| KX225492 | | | |
| KX225493 | | | |
| KX380796 | | | |
| KX380797 | | | |
| KX380798 | | | |
| KX380799 | | | |
| KX380800 | | | |
| KX380801 | | | |
| KX380802 | | | |
| KX380803 | | | |
| KX380804 | | | |
| KX380805 | | | |
| KX380806 | | | |
| KX452051 | | | |
| KX452052 | | | |
| KX452053 | | | |
| KX452054 | | | |
| KX452055 | | | |
| KX452057 | | | |
| KX452058 | | | |
| KX452059 | | | |
| KX452060 | | | |
| KX452061 | | | |
| KX452064 | | | |
| KX452065 | | | |
| KX452067 | | | |
| KX452068 | | | |
| KX458014 | | | |
| KX459386 | | | |
| KX459387 | | | |

| | | | |
|---|---|---|---|
| KX459388 | | | |
| KX459389 | | | |
| KX459390 | | | |
| KX459391 | | | |
| KX459392 | | | |
| KX595191 | | | |
| KX618705 | | | |
| KX618706 | | | |
| KX620451 | | | |
| KX620452 | | | |
| KX620453 | | | |
| KX620454 | | | |
| KX620455 | | | |
| KX621249 | | | |
| KX621250 | | | |
| KX621253 | | | |
| KX951689 | | | |
| KY057365 | | | |
| KY057366 | | | |
| KY057367 | | | |
| KY057368 | | | |
| KY057370 | | | |
| KY057371 | | | |
| KY496854 | | | |
| KY496855 | | | |
| KY496856 | | | |
| KY849724 | | | |
| KY849725 | | | |
| KY849728 | | | |
| KY849730 | | | |
| KY849732 | | | |
| KY849746 | | | |
| KY921902 | | | |
| KY921903 | | | |
| LC011945 | | | |
| LC011946 | | | |
| LC011947 | | | |
| LC011948 | | | |
| LC011949 | | | |
| LC128301 | | | |
| LC335871 | | | |
| LC335872 | | | |
| LC335873 | | | |

| | | | |
|---|---|---|---|
| LC335874 | | | |
| LC335875 | | | |
| LC335876 | | | |
| LC335877 | | | |
| LC335878 | | | |
| LC335879 | | | |
| LC335880 | | | |
| LC335881 | | | |
| LC410183 | | | |
| MF033196 | | | |
| MF033197 | | | |
| MF033198 | | | |
| MF033199 | | | |
| MF033200 | | | |
| MF033201 | | | |
| MF033202 | | | |
| MF033203 | | | |
| MF033204 | | | |
| MF033205 | | | |
| MF033206 | | | |
| MF033207 | | | |
| MF033208 | | | |
| MF033209 | | | |
| MF033210 | | | |
| MF033211 | | | |
| MF033212 | | | |
| MF033213 | | | |
| MF033214 | | | |
| MF033215 | | | |
| MF033216 | | | |
| MF033217 | | | |
| MF033218 | | | |
| MF033219 | | | |
| MF033220 | | | |
| MF033221 | | | |
| MF033222 | | | |
| MF033223 | | | |
| MF033224 | | | |
| MF033225 | | | |
| MF033226 | | | |
| MF033227 | | | |
| MF033228 | | | |
| MF033229 | | | |

| | | | |
|---|---|---|---|
| MF033230 | | | |
| MF033231 | | | |
| MF033232 | | | |
| MF033233 | | | |
| MF033234 | | | |
| MF033235 | | | |
| MF033236 | | | |
| MF033237 | | | |
| MF033238 | | | |
| MF033239 | | | |
| MF033240 | | | |
| MF033241 | | | |
| MF033242 | | | |
| MF033243 | | | |
| MF033244 | | | |
| MF033245 | | | |
| MF033246 | | | |
| MF033247 | | | |
| MF033248 | | | |
| MF033249 | | | |
| MF033250 | | | |
| MF033251 | | | |
| MF033252 | | | |
| MF033253 | | | |
| MF033254 | | | |
| MF033255 | | | |
| MF033256 | | | |
| MF033257 | | | |
| MF033258 | | | |
| MF033259 | | | |
| MF033260 | | | |
| MF033261 | | | |
| MF314188 | | | |
| MF405201 | | | |
| MF681692 | | | |
| MF681693 | | | |
| MF683116 | | | |
| MF683117 | | | |
| MG560265 | | | |
| MG560266 | | | |
| MG560267 | | | |
| MG560268 | | | |
| MG560269 | | | |

| | | | |
|---|---|---|---|
| MG679800 | | | |
| MG679801 | | | |
| MG767211 | | | |
| MG877554 | | | |
| MG877556 | | | |
