# Supplementary material for: Genomic epidemiological characteristics of dengue fever in Guangdong province, China from 2013 to 2017
Source: PLoS Negl Trop Dis. 2020 Mar 3;14(3):e0008049. doi: 10.1371/journal.pntd.0008049 (PMC7053713; doi:10.1371/journal.pntd.0008049)
Supplement: S3 Table — (PDF) [file pntd.0008049.s007.pdf]

S3 Table. Recombinant strains information

Recombinant strains	Breakpoint position		Detection Methods						
	Begin	End	R	G	B	M	C	S	3
D1|D16164|China/GDzs|2016|Viet Nam	4991	6130	9.68E-69	1.16E-59	1.77E-20	1.19E-18	2.85E-19	3.77E-14	4.99E-10
D1|D17026|China/GDfs|2017|Malaysia	589	837	3.40E-26	1.05E-25	6.59E-13	4.74E-02	4.62E-02	1.24E-03	4.99E-10
D1|D16165|China/GDzs|2016|Indonesia	522	883	1.07E-13	3.51E-06	5.45E-11	3.34E-02	2.35E-02	NS	5.96E-07
D2|D16158|China/GDqy|2016	45	9109	4.13E-03	NS	5.41E-03	4.30E-04	4.06E-04	9.15E-44	1.20E-30
D2|D17029|China/GDsz|2017	512	616	2.26E-09	7.22E-06	1.43E-06	NS	NS	NS	1.24E-03
D2|D17029|China/GDsz|2017	7506	7581	NS	3.39E-10	5.16E-05	NS	NS	NS	5.17E-05
D2|D16168|China/GDzs|2016|Philippines	498	603	4.46E-06	3.70E-07	6.87E-05	NS	NS	NS	9.51E-04
D2|D16003|China/GDzh|2016|Thailand	3308	5352	NS	NS	NS	3.30E-02	1.57E-04	NS	1.42E-07
D2|D151665|China/GD|2015	4689	4724	NS	3.36E-04	NS	NS	NS	NS	2.08E-02
D2|D16032|China/GDfs|2016	929	1686	1.65E-04	2.75E-04	1.67E-04	1.46E-03	5.77E-03	NS	NS
D2|D15030|China/GDcz|2015|Malaysia	424	676	1.36E-03	1.14E-02	NS	NS	NS	NS	NS
D2|D17038|China/GDzh|2017	965	1452	2.83E-02	8.21E-03	4.40E-02	6.46E-03	3.52E-03	NS	NS
D3|D151448|China/GDsz|2015|Philippines	9031	9401	1.29E-17	6.55E-07	1.84E-08	3.03E-05	4.44E-06	NS	6.39E-11
D3|D16007|China/GDdg|2016|Malaysia	3293	3413	3.36E-03	2.58E-02	NS	NS	NS	NS	NS
D3|D16007|China/GDdg|2016|Malaysia	6187	6246	1.60E-17	3.23E-16	1.72E-06	6.58E-03	6.21E-03	1.67E-04	1.30E-08
D3|D151440|China/GDjm|2015|Philippines	7965	8005	2.70E-08	1.68E-07	NS	NS	NS	NS	2.21E-03
D3|D16021|China/GDsz|2016|Indonesia	4574	4612	NS	NS	NS	4.20E-02	NS	1.78E-04	NS
D3|D151447|China/GDsz|2015|Thailand	7079	7127	2.49E-06	2.94E-05	NS	NS	NS	NS	NS
D4|D151602|China/GDjm|2015	1	189	1.09E-10	1.25E-09	1.00E-10	NS	NS	NS	3.66E-03
D4|D151602|China/GDjm|2015	3539	4791	2.12E-84	1.48E-81	5.83E-84	3.94E-22	3.83E-22	7.15E-26	3.48E-11
D4|D151602|China/GDjm|2015	6444	6497	4.00E-10	4.85E-05	NS	9.07E-03	8.04E-03	NS	2.55E-05
D4|D151453|China/GDsz|2015|Philippines	1	374	3.08E-32	9.48E-31	2.50E-32	1.46E-07	1.33E-07	6.23E-08	3.48E-11
D4|D151453|China/GDsz|2015|Philippines	3156	3982	1.42E-54	4.51E-52	1.52E-49	2.07E-16	7.58E-17	3.00E-16	3.48E-11
D4|D151453|China/GDsz|2015|Philippines	5212	6358	6.88E-47	5.48E-44	3.81E-47	1.03E-16	1.17E-06	3.12E-12	3.48E-11
D4|D151453|China/GDsz|2015|Philippines	9435	9551	2.57E-15	4.19E-14	1.16E-11	2.07E-02	0.012548	NS	6.96E-07
D4|D151435|China/GDyj|2015|Cambodia	4300	4748	6.85E-06	6.76E-05	6.52E-06	NS	NS	NS	NS
D4|D151435|China/GDyj|2015|Cambodia	5794	6260	8.85E-07	4.24E-08	3.70E-08	NS	NS	2.50E-02	1.28E-03
D4|D15312|China/GDgz|2015|Philippines	1058	1092	3.80E-04	2.99E-05	NS	NS	NS	NS	NS
D4|D15312|China/GDgz|2015|Philippines	7328	7363	2.33E-05	3.23E-04	NS	NS	NS	NS	NS

R: RDP G: GENECONV B: BootScan M: MaxChi C: Chimaera S: SiScan 3: 3Seq

NS: No significant p-value was recorded
