# Supplementary material for: Genomic epidemiological characteristics of dengue fever in Guangdong province, China from 2013 to 2017
Source: PLoS Negl Trop Dis. 2020 Mar 3;14(3):e0008049. doi: 10.1371/journal.pntd.0008049 (PMC7053713; doi:10.1371/journal.pntd.0008049)
Supplement: S4 Table — (PDF) [file pntd.0008049.s008.pdf]

S4 Table. GenBank accession of DENV sequences in this study

| | |
|---|---|
| D1_D15446_China-GDcz_2015 | MN018285 |
| D1_D16152_China-GDcz_2016 | MN018286 |
| D1_D14002_China-GDdg_2014 | MN018287 |
| D1_D16001_China-GDdg-Brazil_2016 | MN018288 |
| D1_D16009_China-GDdg-Malaysia_2016 | MN018289 |
| D1_D16216_China-GDdg-Philippines_2016 | MN018290 |
| D1_D17018_China-GDdg-Thailand_2017 | MN018291 |
| D1_D17037_China-GDdg-Malaysia_2017 | MN018292 |
| D1_D14001_China-GDfs_2014 | MN018293 |
| D1_D14173_China-GDfs_2014 | MN018294 |
| D1_D15025_China-GDfs_2015 | MN018295 |
| D1_D15121_China-GDfs_2015 | MN018296 |
| D1_D17026_China-GDfs-Malaysia_2017 | MN018297 |
| D1_D17103_China-GDfs_2017 | MN018298 |
| D1_D14063_China-GDgz_2014 | MN018299 |
| D1_D14127_China-GDgz_2014 | MN018300 |
| D1_D14223_China-GDgz_2014 | MN018301 |
| D1_D15014_China-GDgz_2015 | MN018302 |
| D1_D15305_China-GDgz-SriLanka_2015 | MN018303 |
| D1_D15308_China-GDgz-Malaysia_2015 | MN018304 |
| D1_D16017_China-GDgz_2016 | MN018305 |
| D1_D17090_China-GDgz_2017 | MN018306 |
| D1_D15349_China-GDhz-Viet_Nam_2015 | MN018307 |
| D1_D14019_China-GDjm_2014 | MN018308 |
| D1_D14088_China-GDmm_2014 | MN018309 |
| D1_D14156_China-GDqy_2014 | MN018310 |
| D1_D15122_China-GDsd_2015 | MN018311 |
| D1_D15123_China-GDsd_2015 | MN018312 |
| D1_D15125_China-GDsd_2015 | MN018313 |
| D1_D16008_China-GDsd-Malaysia_2016 | MN018314 |
| D1_D15340_China-GDst_2015 | MN018315 |
| D1_D15350_China-GDst_2015 | MN018316 |
| D1_D16048_China-GDst_2016 | MN018317 |
| D1_D16061_China-GDst-Philippines_2016 | MN018318 |
| D1_D15009_China-GDsz-Malaysia_2015 | MN018319 |
| D1_D15126_China-GDsz_2015 | MN018320 |
| D1_D15334_China-GDsz-Indonesia_2015 | MN018321 |
| D1_D15335_China-GDsz_2015 | MN018322 |
| D1_D16025_China-GDsz-Bali_2016 | MN018323 |
| D1_D14068_China-GDyf_2014 | MN018324 |
| D1_D14016_China-GDyj_2014 | MN018325 |

| | |
|---|---|
| D1_D17112_China-GDzj_2017 | MN018326 |
| D1_D17510_China-GDzj_2017 | MN018327 |
| D1_D14117_China-GDzs_2014 | MN018328 |
| D1_D15033_China-GDzs_2015 | MN018329 |
| D1_D15045_China-GDzs_2015 | MN018330 |
| D1_D15046_China-GDzs_2015 | MN018331 |
| D1_D16163_China-GDzs-Viet_Nam_2016 | MN018332 |
| D1_D16164_China-GDzs-Viet_Nam_2016 | MN018333 |
| D1_D16165_China-GDzs-Bali_2016 | MN018334 |
| D1_D16166_China-GDzs-Indonesia_2016 | MN018335 |
| D1_D16167_China-GDzs-Malaysia_2016 | MN018336 |
| D2_D15030_China-GDcz-Malaysia_2015 | MN018337 |
| D2_D15044_China-GDcz_2015 | MN018338 |
| D2_D14005_China-GDfs_2014 | MN018339 |
| D2_D16032_China-GDfs_2016 | MN018340 |
| D2_D17025_China-GDfs-Maldives_2017 | MN018341 |
| D2_D17006_China-GDgz-Maldives_2017 | MN018342 |
| D2_D17007_China-GDgz-Thailand_2017 | MN018343 |
| D2_D17013_China-GDgz-Thailand_2017 | MN018344 |
| D2_D15341_China-GDmm-Myanmar_2015 | MN018345 |
| D2_D16005_China-GDmm-Malaysia_2016 | MN018346 |
| D2_D16158_China-GDqy_2016 | MN018347 |
| D2_D16033_China-GDsd_2016 | MN018348 |
| D2_D15023_China-GDsg-Indonesia_2015 | MN018349 |
| D2_D16047_China-GDst-Philippines_2016 | MN018350 |
| D2_D15024_China-GDsw-New_Guinea_2015 | MN018351 |
| D2_D15328_China-GDsz-Taiwan_2015 | MN018352 |
| D2_D16027_China-GDsz_2016 | MN018353 |
| D2_D17027_China-GDsz_2017 | MN018354 |
| D2_D17029_China-GDsz_2017 | MN018355 |
| D2_D14070_China-GDyf_2014 | MN018356 |
| D2_D14003_China-GDzh_2014 | MN018357 |
| D2_D16003_China-GDzh-Thailand_2016 | MN018358 |
| D2_D17038_China-GDzh_2017 | MN018359 |
| D2_D17040_China-GDzh_2017 | MN018360 |
| D2_D17024_China-GDzq-Malaysia_2017 | MN018361 |
| D2_D14115_China-GDzs_2014 | MN018362 |
| D2_D16161_China-GDzs-New_Guinea_2016 | MN018363 |
| D2_D16168_China-GDzs-Philippines_2016 | MN018364 |
| D2_D151663_China-GD-Maldives_2015 | MN018365 |
| D2_D151665_China-GD-Viet_Nam_2015 | MN018366 |
| D3_D15118_China-GDfs_2015 | MN018367 |
| D3_D13056_China-GDzs_2013 | MN018368 |

| | |
|---|---|
| D3_D13146_China-GDfs_2013 | MN018369 |
| D3_D15037_China-GDzs-Indonesia_2015 | MN018370 |
| D3_D15043_China-GDfs_2015 | MN018371 |
| D3_D16007_China-GDdg-Malaysia_2016 | MN018372 |
| D3_D16006_China-GDsd-Indonesia_2016 | MN018373 |
| D3_D151436_China-GDyj_2015 | MN018374 |
| D3_D151655_China-GD_2015 | MN018375 |
| D3_D151658_China-GD_2015 | MN018376 |
| D3_D151660_China-GD_2015 | MN018377 |
| D3_D151447_China-GDsz-Thailand_2015 | MN018378 |
| D3_D151448_China-GDsz-Philippines_2015 | MN018379 |
| D3_D151440_China-GDjm-Philippines_2015 | MN018380 |
| D3_D16016_China-GDsd-Malaysia_2016 | MN018381 |
| D3_D16021_China-GDsz-Bali_2016 | MN018382 |
| D3_D14014_China-GDdg-Indonesia_2014 | MN018383 |
| D3_D16046_China-GDsz_2016 | MN018384 |
| D3_D16154_China-GDsd-India_2016 | MN018385 |
| D3_D13054_China-GDzs_2013 | MN018386 |
| D3_D17139_China-GDdg_2017 | MN018387 |
| D3_D17641_China-GDdg_2017 | MN018388 |
| D3_D17011_China-GDsz-Philippines_2017 | MN018389 |
| D4_D13467_China-GDsz_2013 | MN018390 |
| D4_D15047_China-GDfs-Cambodia_2015 | MN018391 |
| D4_D15299_China-GDyj-Cambodia_2015 | MN018392 |
| D4_D15312_China-GDgz-Philippines_2015 | MN018393 |
| D4_D151435_China-GDyj-Cambodia_2015 | MN018394 |
| D4_D151453_China-GDsz-Philippines_2015 | MN018395 |
| D4_D151602_China-GDjm-Thailand_2015 | MN018396 |
| D4_D16004_China-GDst-Viet_Nam_2016 | MN018397 |
| D4_D16039_China-GDdg_2016 | MN018398 |
